# Oyster Versatile IKKα/βs Are Involved in Toll-Like Receptor and RIG-I-Like Receptor Signaling for Innate Immune Response

**DOI:** 10.3389/fimmu.2019.01826

**Published:** 2019-07-31

**Authors:** Baoyu Huang, Linlin Zhang, Fei Xu, Xueying Tang, Li Li, Wei Wang, Mingkun Liu, Guofan Zhang

**Affiliations:** ^1^Key Laboratory of Experimental Marine Biology, Institute of Oceanology, Chinese Academy of Sciences, Qingdao, China; ^2^Laboratory for Marine Biology and Biotechnology, Qingdao National Laboratory for Marine Science and Technology, Qingdao, China; ^3^National & Local Joint Engineering Laboratory of Ecological Mariculture, Qingdao, China; ^4^Center for Ocean Mega-Science, Chinese Academy of Sciences, Qingdao, China; ^5^Laboratory for Marine Fisheries and Aquaculture, Qingdao National Laboratory for Marine Science and Technology, Qingdao, China

**Keywords:** *Crassostrea gigas*, innate immunity, IKK**α**, IKK**β**, Toll-like receptor, RIG-I-like receptor, NF-**κ**B, interferon regulatory factor

## Abstract

IκB kinases (IKKs) play critical roles in innate immunity through signal-induced activation of the key transcription factors nuclear factor-κB (NF-κB) and interferon regulatory factors (IRFs). However, studies of invertebrate IKK functions remain scarce. In this study, we performed phylogenetic analysis of IKKs and IKK-related kinases encoded in the Pacific oyster genome. We then cloned and characterized the oyster *IKK*α*/*β*-2* gene. We found that oyster IKKα/β-2, a homolog of human IKKα/IKKβ, responded to challenge with lipopolysaccharide (LPS), peptidoglycan (PGN), and polyinosinic-polycytidylic acid [poly(I:C)]. As a versatile immune molecule, IKKα/β-2 activated the promoters of *NF-*κ*B, TNF*α, and *IFN*β, as well as IFN-stimulated response element (ISRE)-containing promoters, initiating an antibacterial or antiviral immune state in mammalian cells. Importantly, together with the cloned oyster IKKα/β-1, we investigated the signal transduction pathways mediated by these two IKKα/β proteins. Our results showed that IKKα/β-1 and IKKα/β-2 could interact with the oyster TNF receptor-associated factor 6 (TRAF6) and that IKKα/β-2 could also bind to the oyster myeloid differentiation factor 88 (MyD88) protein directly, suggesting that oyster IKKα/βs participate in both RIG-I-like receptor (RLR) and Toll-like receptor (TLR) signaling for the reception of upstream immune signals. The fact that IKKα/β-1 and IKKα/β-2 formed homodimers by interacting with themselves and heterodimers by interacting with each other, along with the fact that both oyster IKKα/β proteins interacted with NEMO protein, indicates that oyster IKKα/βs and the scaffold protein NEMO form an IKK complex, which may be a key step in phosphorylating IκB proteins and activating NF-κB. Moreover, we found that oyster IKKα/βs could interact with IRF8, and this may be related to the IKK-mediated activation of ISRE promotors and their involvement in the oyster “interferon (IFN)-like” antiviral pathway. Moreover, the expression of oyster IKKα/β-1 and IKKα/β-2 may induce the phosphorylation of IκB proteins to activate NF-κB. These results reveal the immune function of oyster IKKα/β-2 and establish the existence of mollusk TLR and RLR signaling mediated by IKKα/β proteins for the first time. Our findings should be helpful in deciphering the immune mechanisms of invertebrates and understanding the development of the vertebrate innate immunity network.

## Introduction

The innate immune response represents the first line of defense of eukaryotic organisms against microbial infections (1). In higher vertebrates such as mammals, innate immune signaling begins with pattern recognition receptors (PRRs), such as Toll-like receptors (TLRs) and RIG-I-like receptors (RLRs), which recognize pathogen-associated molecule patterns (PAMPs) (2, 3). This recognition triggers immune signaling cascades, leading to the activation of the key transcription factors NF-κB and interferon regulatory factors (IRFs) and initiating antiviral or antibacterial immune responses (4–6).

The canonical IKKs (IKKα and IKKβ) and IKK-related kinases [TANK-binding kinase 1 (TBK1) and IKKε] play crucial roles in both NF-κB and IRF signaling (7). IKKα and IKKβ are considered the most important inhibitors of NF-κB (IκB) kinases (8). Both proteins have been cloned and purified on the basis of their ability to phosphorylate IκB proteins and activate NF-κB through the canonical IKK-dependent pathway (9, 10). IKK complex assembly is crucial in the IKK-dependent pathway. IKKα and IKKβ are present in cells as part of this high-molecular-weight complex, acting as the catalytic subunits (11, 12). The IKK complex also contains a scaffold and regulatory subunit termed IKKγ or NF-κB essential modulator (NEMO). NEMO is a non-catalytic regulatory subunit originally identified as an essential component of the IKK complex (13, 14). Besides the ability to activate NF-κB, it has also been reported that IKKα can associate with and phosphorylate IRF7 and participate in TLR7/9-induced IFN-α production (15). The IKK-related kinases TBK1 and IKKε have been identified with sequence similarity to IKKα and IKKβ (16, 17). In contrast to IKKα and IKKβ, which activate NF-κB, TBK1, and IKKε play important roles by activating other transcription factors, namely IRF3 and IRF7 (18, 19). The phosphorylation of IRF3 and IRF7 by TBK1 and IKKε promotes IRF3 and IRF7 homodimerization and their subsequent nuclear import, followed by the induction of type I IFN gene expression.

There have been fewer functional studies on IKKs and IKK-related kinases in invertebrates than in vertebrates. In *Drosophila melanogaster*, the catalytic subunit immune-response deficient 5 (IRD5, the fly homolog of mammalian IKKβ) and the regulatory subunit Kenny (the fly homolog of mammalian IKKγ) take part in the immune deficiency (IMD) pathway and form a signaling complex that is thought to tag *Drosophila* Relish for its subsequent cleavage and the activation of antibacterial immune response genes (20, 21). Moreover, IKKε was also identified in *D. melanogaster* and found to participate in regulating the non-apoptotic function of caspases via the degradation of inhibitor of apoptosis proteins (IAPs) (22). Regarding other invertebrates, IKKs and IKK-related kinases have also been cloned from several species, including an IKK homolog from *Pinctada fucata* (23); IKKβ, IKKε1, and IKKε2 from *Scylla paramamosain* (24); and IKKβ and IKKε from *Litopenaeus vannamei* (25). Such studies are helpful for understanding the functions of invertebrate IKKs. However, whether invertebrate IKKs are involved in RLR or TLR signaling to activate antiviral or antibacterial cytokines and the details of the associated signaling transduction pathways are largely unknown and require further investigation.

The Pacific oyster (*Crassostrea gigas*) is a representative bivalve mollusk and lophotrochozoan protostome (26). As a sessile filter feeder that lives in the estuary and intertidal zone, the oyster is frequently exposed to a large variety of pathogens, making it an attractive model for studying the innate immune system of invertebrates. Moreover, oysters are distributed worldwide and support major aquaculture and fishery industries worldwide (27); however, recently, oyster mass mortality caused by viruses or bacteria has severely affected oyster production (28–31). Hence, there is an urgent need to better understand the immune mechanisms of oysters in order to promote the development of new strategies for controlling such diseases.

Efforts have been made to elucidate the mechanisms of innate immunity in oysters, and some progress has been made. Of note, the oyster genome is predicted to encode several evolutionarily conserved nucleic acid sensors and their downstream signaling molecules (32, 33). Additionally, the conserved RLR and TLR innate immune signaling pathways have been preliminarily demonstrated in oysters, with studies reporting the existence of the RIG-I-mitochondrial antiviral (MAVS) signaling protein and TLR-MyD88 signaling axis in the oyster (34–36). Moreover, *IKK-like* and *TBK1* genes have been identified in the Pacific oyster (37, 38), as well as a *NEMO* gene (39). To date, three IκB genes have also been identified in oyster (40, 41). However, the functions of oyster IKK genes involved in innate immune signaling should be further investigated in greater detail.

In this study, we performed phylogenetic analysis of all IKKs and IKK-related kinases encoded in the oyster genome, and we subsequently cloned and characterized IKKα/β-2 from oyster. In addition, we focused on the immune signaling transduction pathways mediated by IKKα/β-1 and IKKα/β-2 and established the presence of rudimentary oyster TLR and RLR signaling. Our results should be useful for further research into the immune mechanisms of invertebrates and the development of disease-resistant strategies.

## Materials and Methods

### Phylogenetic Analysis and Classification of IKK Sequences

The IKK sequences used for the alignment and phylogenetic analysis were downloaded from the NCBI database (https://www.ncbi.nlm.nih.gov/), and the eukaryotic translation initiation factor 2-alpha kinase 4 (EIF2AK4) sequence was used as the outgroup in phylogenetic analyses. Alignment of all sequences was conducted using MAFFT 7.221 software (42) with the E-INS-I algorithm. Phylogenetic analysis was then performed with the LG + Gamma + Invariant evolution model using RAxML software (43). The consistency test was performed with 1,000 repetitions using the bootstrap method.

### Cloning and Sequence Analysis of Oyster *IKKα/β* Genes

An oyster cDNA library was first prepared. Total RNA was extracted from oyster gill and mantle samples using TRIzol Reagent (Invitrogen, USA) and then treated with DNase I (Promega, USA). First-strand cDNA synthesis using the treated RNA as a template was performed using Promega M-MLV reverse transcriptase according to the manufacturer's instructions. Then, according to information from the *C. gigas* genome (32) and the sequences deposited in the GenBank (No. NM_001308886.1 and XM_011450699.2), oyster *IKK*α*/*β*-1* and *IKK*α*/*β*-2* gene sequences were amplified using specific primers (Supplementary Table 1). The PCR products were purified using the E.Z.N.A Gel Extraction Kit (OMEGA, USA) and cloned into the pMD19-T vector (Takara, Japan). The recombinant vectors were transformed into *E. coli Trans*1-T1 competent cells (Transgen, China) and sequenced (Sangon Biotech, China).

Open Reading Frame Finder (http://www.ncbi.nlm.nih.gov/gorf/orfig.cgi) was used to analyze cDNA sequences and deduce the corresponding polypeptides they encode. The Simple Modular Architecture Research Tool (SMART; http://smart.emblheidelberg.de) was used to predict protein domains. Protein sequences from different species were downloaded from NCBI (http://www.ncbi.nlm.nih.gov/guide/proteins/) and compared using the ClustalW2 program (http://www.ebi.ac.uk/Tools/msa/clustalw2/). The calculated molecular mass and the theoretical isoelectric point (pI/Mw) were analyzed by the ExPASy compute pI/Mw tool (https://web.expasy.org/compute_pi/).

### Animals and Immune Challenge

Healthy oysters with an average shell height of 60 mm were collected from a farm in Qingdao, Shandong Province, China. All animal experiments were conducted in accordance with the guidelines and approval of the respective Animal Research and Ethics Committees of the Chinese Academy of Sciences. Experimental specimens were acclimatized in aerated and filtered seawater at 22 ± 0.5°C for more than 1 week prior to the execution of experiments. Samples of the gonad, muscle, blood, mantle, gills, labial palps, and digestive gland were collected from three oysters and snap-frozen in liquid nitrogen for analysis of tissue-specific expression patterns.

To examin*e CgIKK*α*/*β*-2* expression patterns after challenge with lipopolysaccharide (LPS), peptidoglycan (PGN) and polyinosinic-polycytidylic acid [poly(I:C)], 200 oysters were randomly divided into four groups. The oysters of the control group were injected with 100 μL phosphate-buffered saline (PBS, pH = 7.4), while those in the LPS, PGN, and poly(I:C) experimental groups were injected with 100 μL of LPS, PGN, or poly(I:C) suspended in PBS at concentrations of 1.0 μg/mL, 1.0 μg/mL, and 1.0 mg/mL, respectively. Hemolymph samples were collected from five oysters per group at 0, 6, 12, 24, 48, and 72 h post-injection. The collected hemolymph was immediately centrifuged at 1,000 × g for 10 min at 4°C in order to harvest hemocytes for RNA preparation. The total RNA was then extracted, the template for quantitative real-time PCR (qRT-PCR) was prepared, and qRT-PCR was performed to analyze the mRNA expression level of *CgIKK*α*/*β*-2* after challenge.

### qRT-PCR Analysis of *CgIKKα/β-2* mRNA Expression

Briefly, qRT-PCR was performed in a 7500 Fast Real-Time PCR System (Applied Biosystems, USA) using a SYBR Green Real Time PCR Master Mix kit (Takara) to quantify mRNA expression levels. The primers used for qRT-PCR analysis are listed in Supplementary Table 1. The β*-actin* (*ACTB*) gene (GenBank No. NM_001308859.1) was employed as an internal control for cDNA normalization. And the relative mRNA expression level of *CgIKK*α*/*β*-2* transcripts was calculated using the comparative Ct method (2^−ΔΔ*Ct*^ method) (44).

### Plasmid Construction, Cell Culture, and Transfection

Dual-luciferase reporter (DLR) assays, Yeast two-hybrid (Y2H) assays, and Co-immunoprecipitation (Co-IP) assays are all need to construct related plasmids. In DLR assays, the open reading frame (ORF) of *CgIKK*α*/*β*-2* was amplified using Phusion High-Fidelity DNA polymerase (Thermo Fisher Scientific, USA) with specific primers (Supplementary Table 1). The plasmids pCMV-Myc (Clontech, USA) was digested with *EcoR*I (New England Biolabs, USA). And the purified PCR products were fused with the purified digested plasmids using the Ligation-Free Cloning System (Applied Biological Materials, Inc., Canada) according to the manufacturer's instructions. The plasmids of *NF-*κ*B, TNF*α, and ISRE reporter genes were purchased from Beyotime Biotechnology Corporation of China, and the plasmids of *IFN*β reporter genes were purchased from Stratagene Company of USA. pRL-CMV *Renilla* luciferase plasmids were purchased from Promega Company of USA. In Y2H assays, the ORF of each gene was also amplified using Phusion High-Fidelity DNA polymerase with specific primers (Supplementary Table 1). The plasmids pGBKT7 and pGADT7 (Clontech) were digested with *EcoR*I and *BamH*I (New England Biolabs). And then the purified PCR products were fused with the purified digested plasmids using the Ligation-Free Cloning System. For Co-IP studies, the plasmids for the -myc fusion protein expression were constructed using the same methods mentioned in DLR assays. For the -flag fusion protein expression, the pCMS-EGFP-FLAG (constructed by our lab) plasmids were digested with *Xho*I (New England Biolabs) and the purified PCR products were fused with the purified digested plasmids using the Ligation-Free Cloning System.

HEK293T cells (ATCC, USA) were cultured in Dulbecco's modified Eagle's medium (high glucose) (HyClone, USA) supplemented with 10% heat-inactivated fetal bovine serum (Gibco, USA) and 1 × penicillin-streptomycin solution (Solarbio, China). Cells were grown in an atmosphere of 95% air/5% CO_2_ at 37°C and subcultured every 3–4 days. Plasmids were transfected into HEK293T cells using Lipofectamine 3000 reagent (Life Technologies, USA) according to the manufacturer's instructions.

### Dual-Luciferase Reporter Assays

Dual-luciferase reporter assays were performed in HEK293T cells to detect the effects of oyster IKKα/β-2 protein on transcription from the *NF-*κ*B, TNF*α, ISRE, and *IFN*β promoters using Myc-fused protein expressing vectors. Briefly, cells in 24-well plates (Corning, USA) were transfected with 0.1 μg of reporter gene plasmids, 0.01 μg of pRL-CMV *Renilla* luciferase plasmid (Promega), and varying amounts of expression plasmids or empty expression vector (as a control). The pRL-CMV *Renilla* luciferase plasmid was used as an internal control. At 24–48 h post-transfection, the Dual-Luciferase Reporter Assays System (Promega) was used to measure the activities of firefly and *Renilla* luciferases according to the manufacturer's instructions. Experiments were performed in triplicate.

### Yeast Two-Hybrid Assays

Yeast two-hybrid (Y2H) assays were performed to detect interactions between proteins. Briefly, using the Clontech Matchmaker Gold Yeast Two-Hybrid System (Takara), the fusion protein expression plasmids pGADT7 (AD vector) and pGBKT7 (BD vector) were transformed into the Y187 and Gold yeast strains, respectively, according to the manufacturer's instructions. Y187 cells were cultured on selective plates with synthetically defined (SD) medium lacking leucine (SD/-Leu), whereas Gold cells were cultured on SD plates lacking tryptophan (SD/-Trp). After 3–5 days, yeast strains able to grow on SD/-Leu and SD/-Trp media were hybridized in 2× yeast extract peptone dextrose (YPDA) medium and selected on double drop-out (SD/-Leu/-Trp) medium. Interactions between proteins were detected based on the ability of the hybridized clones to grow on quadruple drop-out (SD/-Ade/-His/-Leu/-Trp) medium supplemented with X-α-Gal and aureobasidin A (Takara).

### Co-immunoprecipitation (Co-IP) Assays

HEK293T cells were divided between two or more Petri dishes (10-cm diameter, Corning) and cultured for 24 h. Fused pCMV-Myc plasmids were co-transfected with vectors expressing FLAG-tagged fusion proteins or empty FLAG vector (control). After 24–36 h, cells were harvested in cell lysis buffer (Beyotime, China). Input samples were prepared from the cell lysate, and the remaining lysates were mixed with anti-FLAG M2 magnetic beads (Sigma, USA) under gentle shaking on a roller at 4°C for 2–4 h. The beads were then washed three times with cell lysis buffer. Input and Co-IP samples were incubated with 2× protein sodium dodecyl sulfate polyacrylamide gel electrophoresis loading buffer (Takara) at 100°C for 3–5 min. Proteins were analyzed by western blotting using anti-Myc antibody (Roche, Switzerland) and anti-FLAG antibodies (Sigma).

### *In vitro* Protein Dephosphorylation Assay

Transfected HEK293T cells were lysed as described above except that the lysis buffer did not contain any phosphatase inhibitors. Protein dephosphorylation assay was carried out in a 200 μl reaction consisting of 200 μg of cellular protein and 20 units of calf intestinal phosphatase (CIP, Sigma). The reaction was incubated for 1–2 h at 37°C and then subjected to immunoblot analysis.

### Statistical Analysis

Each experiment (*N* = 3) was repeated at least twice. Statistical analysis was performed with Student's *t*-test for the comparison between two groups or by one-way ANOVA followed by LSD multiple group comparisons using the SPSS13.0. Differences were considered significant at *P* < 0.05.

## Results

### Phylogenetic Analysis of IKKs and IKK-Related Kinases in the Oyster Genome

**Figure 1:**
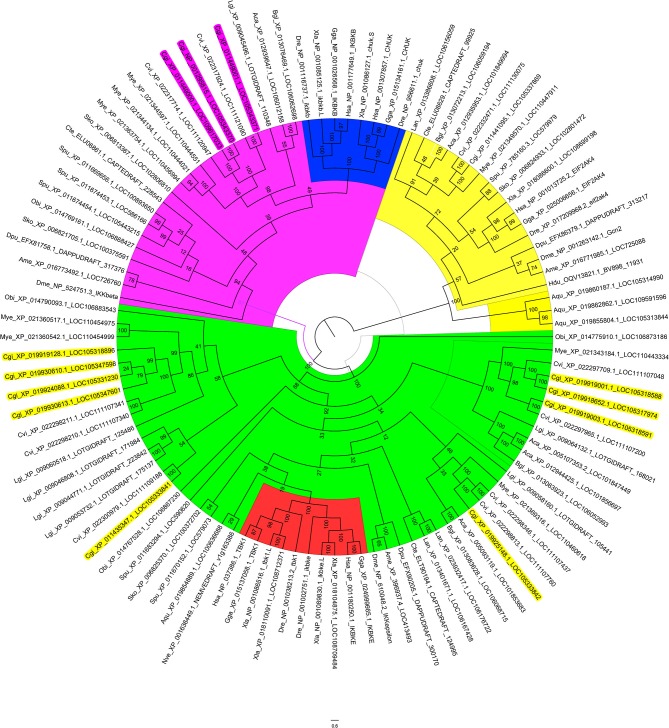
Phylogenetic tree of IKKs and IKK-related kinases from various species. The phylogenetic tree is divided into three branches: in pink are IKKα/IKKβ family members, in green are TBK1/IKKε family members, and in yellow are the outgroup sequences. Blue indicates the vertebrate IKKα and IKKβ branches. Red indicates the vertebrate TBK1/IKKε branches. The names of oyster IKKs and IKK-related kinases are shown in pink and yellow, with pink indicating oyster IKKα/β proteins, and yellow indicating oyster IKKε or IKKε-like proteins.

As the oyster genome has been sequenced (32), we first performed sequence analysis and constructed a phylogenetic tree of the predicted IKKs and IKK-related kinases in the oyster genome (Figure 1). We found that all IKKs or IKK-related kinases in oyster could be classified into two groups: an IKKα/IKKβ group and a TBK1/IKKε group. Three proteins with protein IDs of XP_011449000.1, NP_001295815.1, and XP_011449001.1 belonged to the IKKα (also called Chuk, conserved helix-loop-helix ubiquitous kinase) or IKKβ families. Because the IKKα and IKKβ proteins of vertebrates are first clustered together (shown in blue in Figure 1), it is difficult to determine whether these three oyster IKK proteins are IKKα or IKKβ proteins. Therefore, we named these proteins IKKα/β. The other oyster IKK proteins all belonged to the TBK1/IKKε family.

### Identification and Sequence Analysis of Oyster *IKKα/β-2* Gene

Owing to the crucial roles that IKKα and IKKβ play in innate immunity, in-depth analysis of the three oyster IKK proteins was performed. The sequences of XP_011449000.1 and NP_001295815.1 were identical, and the sequence of this gene has been cloned and verified (37); this will be referred to as *CgIKK*α*/*β*-1* hereafter. The remaining IKK gene (XP_011449001.1) was cloned in the present study and is referred to here as *CgIKK*α*/*β*-2*.

**Figure 2:**
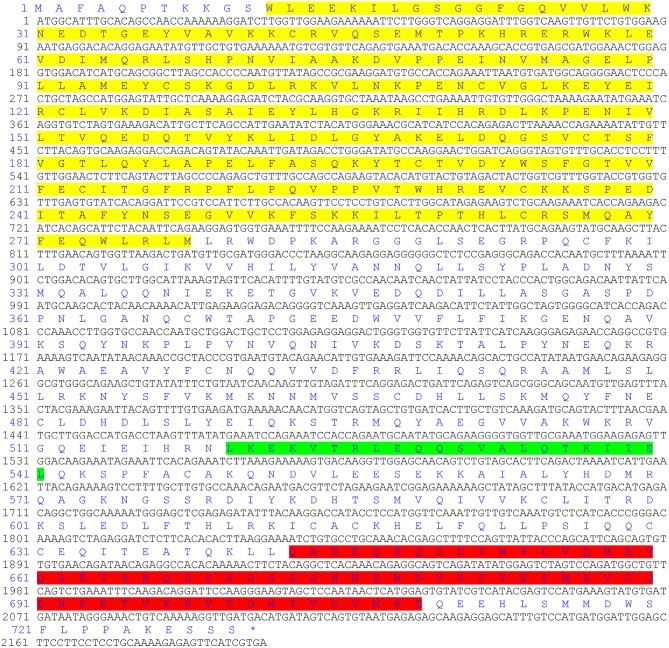
The ORF of CgIKKα/β-2 and its putative protein sequence. Yellow indicates the protein kinase domain, green indicates a leucine zipper, and the C-terminal helix-loop-helical domain is shown in red.

**Figure 3:**
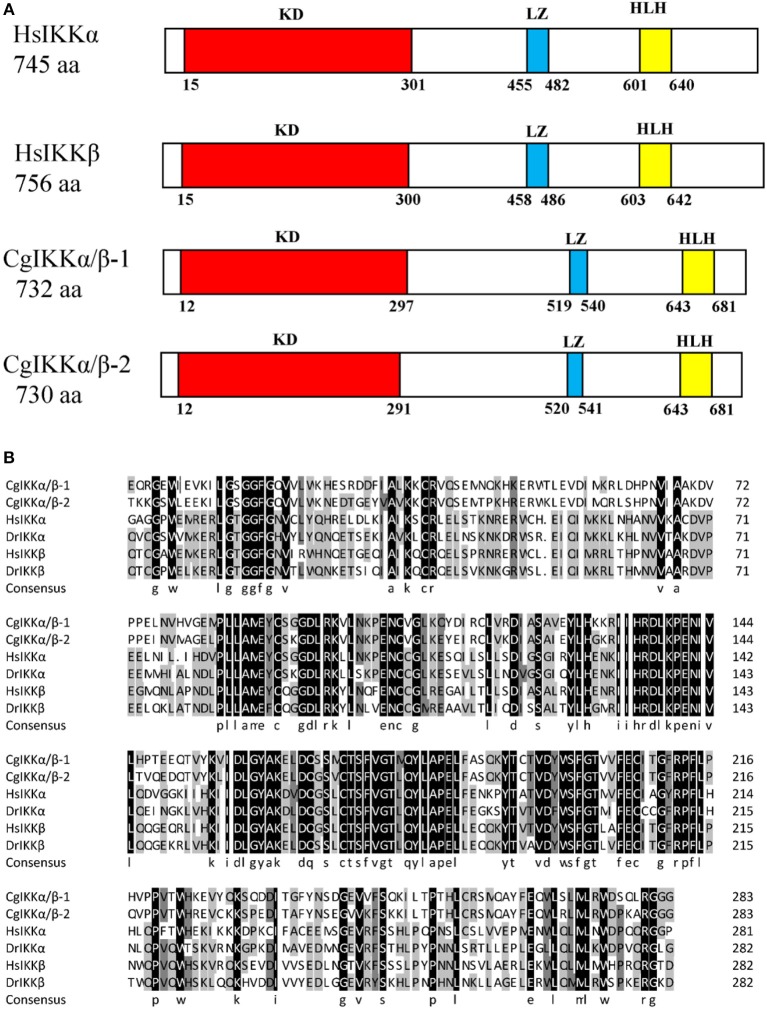
Schematic representation of human and oyster IKKα/β protein structure **(A)** and multiple sequence alignment of IKKα and IKKβ proteins from different species **(B)**. Sequences shaded in black are positions where all the sequences share the same amino acid residue, whereas those shaded in gray and light gray represent conservative and semi-conservative amino acid substitutions, respectively. Hs, *Homo sapiens*; Dr, *Danio rerio*. The GenBank accession numbers for sequences used in this alignment are as the followings: HsIKKα (AAC51662.1), HsIKKβ (AAC64675.1), DrIKKα (NP_956611.1), and DrIKKβ (NP_001116737.1).

The ORF of the *CgIKK*α*/*β*-2* gene is 2193 bp, and it encodes a putative protein of 730 amino acids. The putative protein was estimated to be 83.6 kDa, with a predicted isoelectric point of 8.07. Theoretical molecular weight and specificity of proteins detected for Western Blot analysis in this research were all shown in Supplementary Figure 2. Sequence analysis revealed that the CgIKKα/β-2 amino acid sequence was up to 70% identical to that of the cloned CgIKKα/β-1 protein. The CgIKKα/β-2 protein is predicted to contain a typical N-terminal serine/threonine protein kinase domain, an intermediate leucine zipper, and a C-terminal HLH domain (Figures 2, 3A). The core protein kinase domain is highly conserved (Figure 3B).

### *CgIKK**α**/**β**-2* mRNA Expression in Different Tissues and Response to Challenge

**Figure 4:**
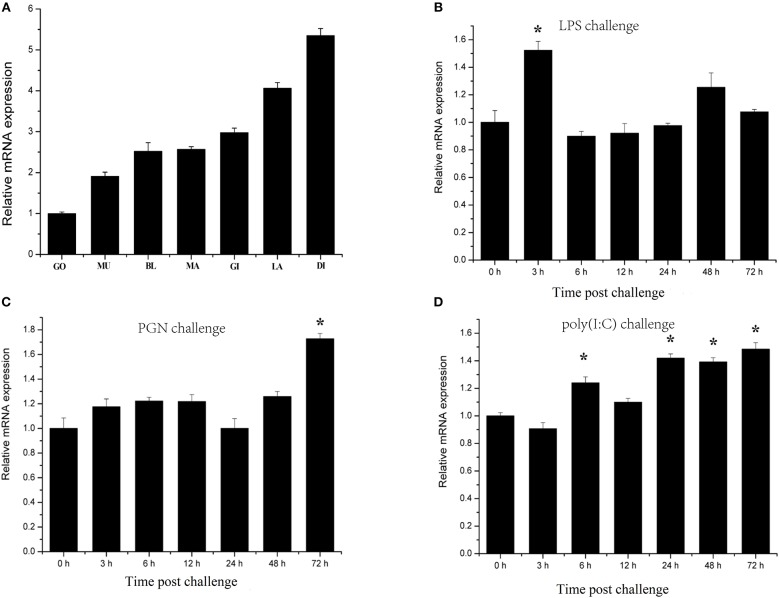
Expression profiles of *CgIKK*α*/*β*-2* mRNA in different tissues **(A)** and following challenge with different stimulants **(B–D)** as determined by qRT-PCR. Tissues: GO, Gonad; MU, Muscle; BL, Blood; MA, Mantle; GI, Gill; LA, Labial palp; DI, Digestive gland. β*-actin* gene expression was used as an internal control, and gonad was used as a reference sample. Vertical bars represent the mean ± SD (*N* = 3). Challenges: the different stimulants is LPS, PGN, and poly(I:C). The expression level of *CgIKK*α*/*β*-2* was determined at 0, 3, 6, 12, 24, 48, and 72 h after challenge. β*-actin* gene expression was used as an internal control, and time 0 h was used as a reference sample. Vertical bars represent the mean ± SD (*N* = 3).

Characterization of transcription responses to challenge is an effective approach for revealing mechanisms of oyster immunity. The tissue distribution of *CgIKK*α*/*β*-2* mRNA was analyzed using qRT-PCR. As shown in Figure 4A, *CgIKK*α*/*β*-2* expression was observed in all tested tissues of *C. gigas*, with the highest expression of *CgIKK*α*/*β*-2* mRNA present in the digestive gland. *CgIKK*α*/*β*-2* expression in the digestive gland was approximately six-fold higher than that in the gonad (Figure 4A).

*CgIKK*α*/*β*-2* temporal expression profiles in response to challenge with various stimulants were analyzed using qRT-PCR. *CgIKK*α*/*β*-2* expression was induced after LPS, PGN, and double-stranded RNA virus analog poly(I:C) challenge, although its expression level did not change drastically (Figures 4B–D).

### CgIKK**α**/**β**-2 Activates ISRE and *NF-**κ**B* Promoters in Mammalian Cells

**Figure 5:**
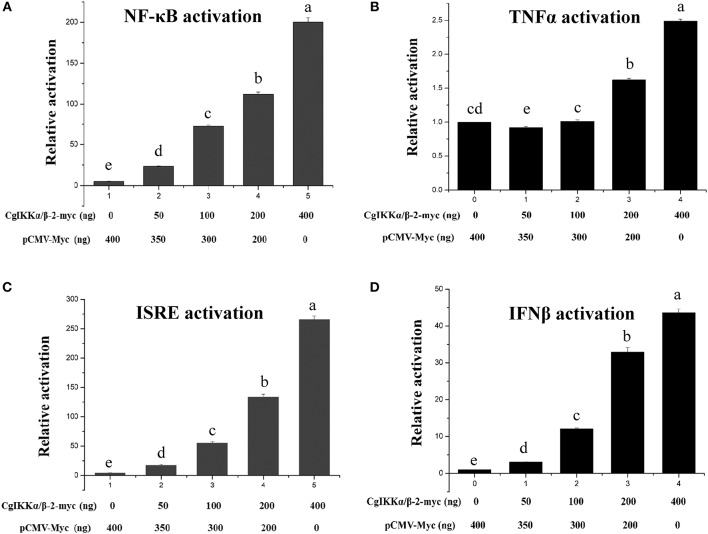
CgIKKα/β-2 activated the *NF-*κ*B* **(A)**, *TNF*α **(B)**, ISRE **(C)**, and *IFN*β **(D)** promoters in a concentration-dependent manner. Activation was detected using dual luciferase reporter assays in human HEK293T cells. Results are displayed as the fold change from the control group. Vertical bars represent the mean ± SD (*N* = 3).

Because IKK family members represent the crucial convergence of upstream signaling and downstream activation of key transcription factors for the final immune response, dual-luciferase reporter assays were employed to determine whether CgIKKα/β-2 activates transcription through *NF-*κ*B, TNF*α, ISRE, and *IFN*β promoters. The expression plasmid pCMV-CgIKKα/β-2, the *NF-*κ*B* (or *TNF*α, ISRE, or *IFN*β) promoter reporter plasmid, and the internal reference plasmid pRL-CMV were co-transfected into HEK293T cells, and luciferase activity was determined after transfection for 24–36 h. The results showed that overexpression of the CgIKKα/β-2 protein activated the *NF-*κ*B, TNF*α, ISRE, and *IFN*β promoters (although the activation of *TNF*α was weak), and this activation was concentration-dependent (Figure 5). These results show that CgIKKα/β-2 is a versatile protein that may participate in multiple immune signaling pathways in oysters.

### CgIKKα/βs Are Involved in Oyster TLR and RLR Signaling

**Figure 6:**
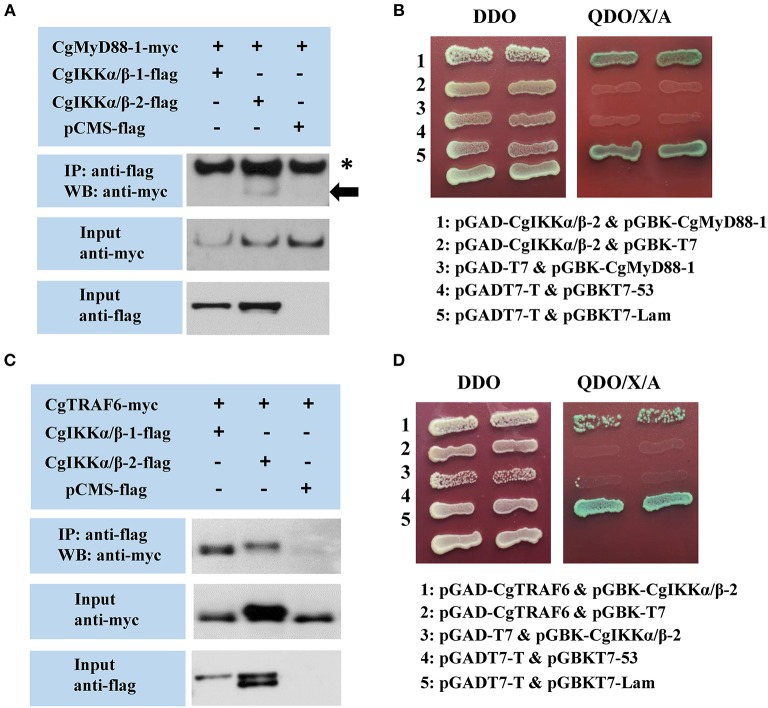
Interactions between oyster IKKα/βs and other proteins detected by co-immunoprecipitation (Co-IP) and yeast two hybrid (Y2H) assays. **(A)** Co-IP results showing that CgMyD88-1 directly interacts with CgIKKα/β-2 but not with CgIKKα/β-1. Asterisk represents the heavy chain of mouse IgG. Anti-Myc western blot bands show the expression of CgMyD88-1-Myc and anti-FLAG western blot bands show the expression of CgIKKα/β-1-FLAG and CgIKKα/β-2-FLAG. **(B)** Interaction between CgIKKα/β-2 and CgMyD88-1 was detected by Y2H assay. DDO, -Leu/-Trp double-dropout media; QDO/X/A, -Ade/-His/-Leu/-Trp quadruple dropout media with X-α-Gal and aureobasidin A. pGADT7-T and pGBKT7-53 were used for the positive control; pGADT7-T and pGBKT7-Lam were used for the negative control. **(C)** Co-IP results showing that CgTRAF6 directly interacts with CgIKKα/β-1 and CgIKKα/β-2. Anti-Myc western blot bands show the expression of CgTRAF6-Myc and anti-FLAG western blot bands show the expression of CgIKKα/β-1-FLAG and CgIKKα/β-2-FLAG. **(D)** Interaction between CgIKKα/β-2 and CgTRAF6 was detected by Y2H assay.

We used Co-IP and Y2H assays to validate the interaction of CgIKKα/β-1 and CgIKKα/β-2 withCgMyD88 and CgMAVS, the key adaptors of oyster TLR and RLR signaling pathways, respectively. Co-transfection of plasmids of CgIKKα/β-1, CgIKKα/β-2, and CgMyD88-1 (GenBank Accession number: KC155821.1) or CgMAVS (GenBank Accession number: KY630189) in HEK293T cells revealed that the oyster CgIKKα/β-2 could bind to CgMyD88-1 (Figures 6A,B) but did not interact directly with CgMAVS (results not shown). However, the Co-IP experimental results showed that both CgIKKα/β-1 and CgIKKα/β-2 could interact with the oyster TRAF6 (Figure 6C), and the interaction between CgIKKα/β-2 and CgTRAF6 was confirmed by Y2H assays (Figure 6D).

### CgIKKα/β-1, CgIKKα/β-2, and CgNEMO Form a Signal Complex

**Figure 7:**
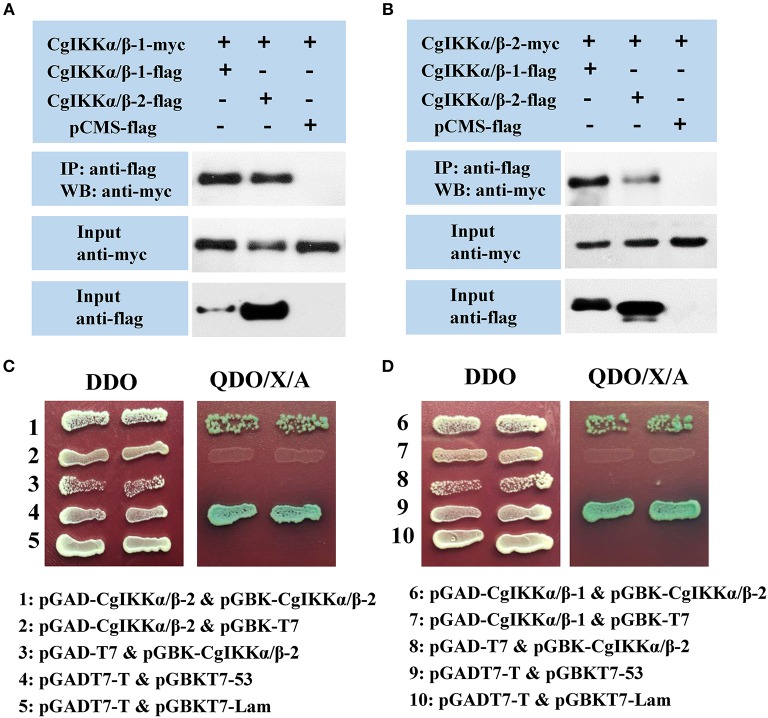
CgIKKα/β-1 and CgIKKα/β-2 each form a homodimer and can form a heterodimer with each other. **(A)** Co-IP results showing that CgIKKα/β-1 and CgIKKα/β-2 interact with CgIKKα/β-1. Anti-Myc western blot bands show the expression of CgIKKα/β-1-Myc and anti-FLAG western blot bands show the expression of CgIKKα/β-1-FLAG and CgIKKα/β-2-FLAG. **(B)** Co-IP results showing that CgIKKα/β-1 and CgIKKα/β-2 interact with CgIKKα/β-2. Anti-Myc western blot bands show the expression of CgIKKα/β-2-Myc and anti-FLAG western blot bands show CgIKKα/β-1-FLAG and CgIKKα/β-2-FLAG. **(C)** Interaction between CgIKKα/β-2 and CgIKKα/β-2 was detected by Y2H assay. **(D)** Interaction between CgIKKα/β-1 and CgIKKα/β-2 was detected by Y2H assay.

**Figure 8:**
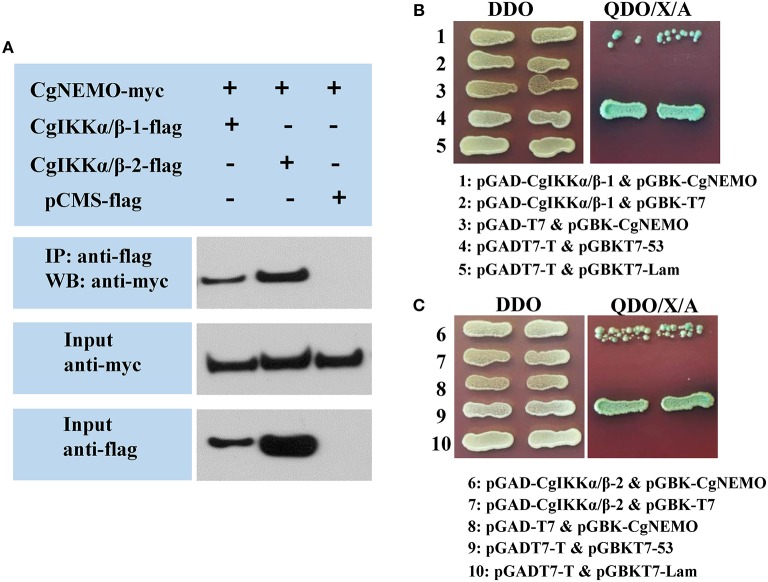
Oyster NEMO interacts with both CgIKKα/β-1 and CgIKKα/β-2. **(A)** Co-IP results showing that CgNEMO directly interacts with CgIKKα/β-1 and CgIKKα/β-2. Anti-Myc western blot bands show the expression of CgNEMO-Myc and anti-FLAG western blot bands show the expression of CgIKKα/β-1-FLAG and CgIKKα/β-2-FLAG. **(B)** Interaction between CgIKKα/β-1 and CgNEMO was confirmed by Y2H assay. **(C)** Interaction between CgIKKα/β-2 and CgNEMO was confirmed by Y2H assay.

As IKKα, IKKβ, and NEMO are the key components of the IKK complex, we examined the interactions and relationships between these three proteins in the oyster. First, Co-IP assay results showed that both CgIKKα/β-1 and CgIKKα/β-2 could form homodimers, as well as bind to each other to form heterodimers (Figures 7A,B). The interactions between CgIKKα/β-2 and CgIKKα/β-2 and between CgIKKα/β-1 and CgIKKα/β-2 were also confirmed by Y2H assays (Figures 7C,D). Next, we test the interaction between CgNEMO and the two CgIKKα/β proteins. The results of Co-IP and Y2H experiments showed that the oyster NEMO protein interacted with both CgIKKα/β-1 and CgIKKα/β-2 (Figure 8A). These interaction results were also confirmed by Y2H assays (Figures 8B,C).

### CgIKKα/β-1 and CgIKKα/β-2 Interact With CgIRF8 and May Phosphorylate Oyster IκB Proteins

**Figure 9:**
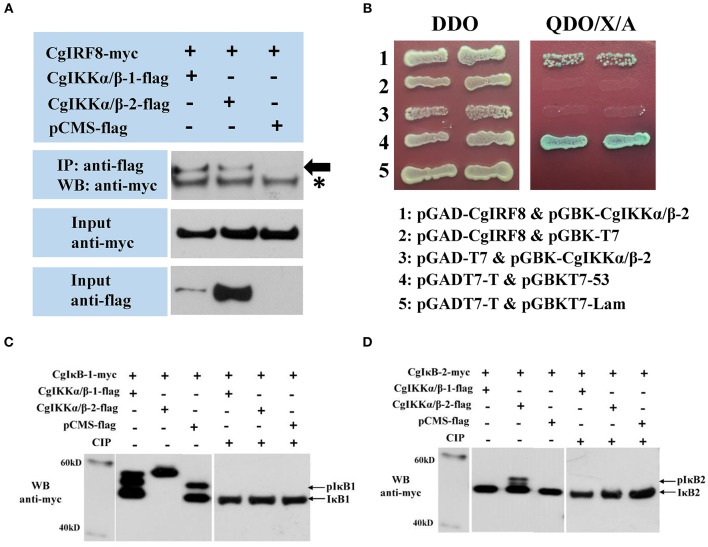
CgIKKα/β-1 and CgIKKα/β-2 recruit oyster IRF8 and may phosphorylate oyster IκB proteins. **(A)** Interaction between CgIKKα/β-1 (or CgIKKα/β-2) and CgIRF8 was detected by Co-IP assays. Anti-Myc western blot bands show the expression of CgIRF8-Myc and anti-FLAG western blot bands show the expression of CgIKKα/β-1-FLAG and CgIKKα/β-2-FLAG. Asterisk represents the heavy chain of mouse IgG. **(B)** Interaction between CgIKKα/β-2 and CgIRF8 was confirmed by Y2H assay. **(C)** CgIKKα/β-1 and CgIKKα/β-2 may phosphorylate oyster IκB1. HEK293T cells were seeded in 6-well plates at 10^7^ cells per well overnight and transfected with the indicated plasmids (1 μg each) for 24 h. The cell lysates (200 μg) were treated with or without CIP (20 U) for 1–2 h at 37°C. Then the lysates were detected by immunoblotting with the anti-Myc antibody. **(D)** CgIKKα/β-2 may phosphorylate oyster IκB2.

Because CgIKKα/β-2 activated ISRE and human *IFN*β reporter genes in a dose-dependent manner, we assessed the relationship between the oyster IKKα/β proteins and CgIRF2 (GenBank Accession number: KY630191) and CgIRF8 (GenBank Accession number: KY630192), which were characterized in our previous study (34). The Co-IP results showed that both CgIKKα/β-1 and CgIKKα/β-2 could interact with CgIRF8 (Figure 9A) but not with CgIRF2 (data not shown). The Y2H results also confirmed the interaction between CgIKKα/β-2 and CgIRF8 (Figure 9B).

Another crucial transcription factor, NF-κB, was also examined by attempting to determine the effect of CgIKKα/βs on the IκBs of oyster. A CgIKKα/β-1 or CgIKKα/β-2 expression vector was co-transfected with oyster IκB protein vectors into HEK293T cells. We found that, in contrast to the control, CgIKKα/β-1 or CgIKKα/β-2 expression resulted in obvious shifts in the CgIκB1 bands detected by western blotting. Additionally, CgIKKα/β-2 expression shifted the CgIκB2 band. In order to verify whether the shifted bands represent the phosphorylated IκBs, we tried to perform a dephosphorylation assay *in vitro*. And the results showed that shifted bands disappeared after being treated with CIP (Supplementary Figure 3; Figures 9C,D).

## Discussion

Animals are constantly threatened by the invasion of various pathogenic microorganisms and have evolved immune defense systems to eliminate such threats. Innate immunity is one of the first lines of defense for animals, mainly relying on the recognition of PAMPs by PRRs and subsequent signal transduction. In contrast to vertebrates, invertebrates lack adaptive immunity and rely on innate immunity alone for host defense. In this report, we focused on the innate immune signaling of the oyster mediated by IKK proteins in order to better understand the innate immune mechanisms of invertebrates.

The Pacific oyster genome sequence not only enables comparative genomic analyses of mollusks but also provides a model species for broad-spectrum genomic studies of shellfish biology (32). Because IKKs and IKK-related kinases represent the crucial convergence of upstream signaling and downstream activation of key transcription factors for the final immune response, we performed phylogenetic analysis of all IKKs and IKK-related kinases encoded in the Pacific oyster genome. These oyster IKKs could be divided into two groups: the IKKα/IKKβ group and TBK1/IKKε group. Interestingly, the IKKα and IKKβ proteins of vertebrates clustered together, which may indicate that the *IKK*α and *IKK*β genes of vertebrates are derived from the same ancestral gene following duplication and functional differentiation. Therefore, as we do not know whether the oyster IKK genes in the IKKα/IKKβ group are *IKK*α or *IKK*β, we named these genes *CgIKK*α*/*β.

In this study, we determined that the three oyster genes belonging to the IKKα/IKKβ family actually encode two proteins, as the sequences of XP_011449000.1 and NP_001295815.1 were identical. The sequence of this gene has been confirmed and was termed *CgIKK*α*/*β*-1* in this study. We cloned the remaining oyster IKKα/IKKβ gene (XP_011449001.1) and named it *CgIKK*α*/*β*-2*. CgIKKα/β-2 is a typical IKK consisting of the typical N-terminal serine/threonine protein kinase domain, an intermediate leucine zipper, and a C-terminal HLH domain. The protein kinase domain is highly conserved, suggesting the functional conservation of the protein.

Mammalian IKKα and IKKβ play important roles in the immune response by activating the transcription factor NF-κB (12). In this study, qRT-PCR showed that the expression of *CgIKK*α*/*β*-2* was ubiquitous in all tissues of the oyster. The universal expression of the *CgIKK*α*/*β*-2* mRNA indicates that *CgIKK*α*/*β*-2* may be essential for most physiological functions in *C. gigas*. Additionally, the expression of *CgIKK*α*/*β*-2* was upregulated after LPS, PGN, and poly(I:C) challenge, indicating that CgIKKα/β-2 may participate in antibacterial and antiviral responses of the host. The induced expression profiles of *CgIKK*α*/*β*-2* after various types of challenge showed the versatility of this gene in oyster innate immunity. This versatility was also confirmed by the results of dual-luciferase assays. Overexpression of the CgIKKα/β-2 protein activated the *NF-*κ*B, TNF*α, ISRE, and *IFN*β promoters (although the activation of *TNF*α was weak) in a concentration-dependent manner. It is reported that the promoter region (2 kb of 5' flanking sequence of the genes) of some key immune genes of oysters, such as the oyster RIG-I-like receptor, contains a large number of NF-κB and IRF binding sites (45). The NF-κB reporter gene plasmids used in the present study exactly contain NF-κB binding sites for detecting NF-κB transcriptional activity levels. And the ISRE reporter gene plasmids used in the present study contained IRF binding sites for detecting IRF transcriptional activity levels. And as shown in Supplementary Figure 1, in the promotor region of some oyster crucial immune genes, such as *TLR, MyD88*, and *IL-17*, lots of NF-κB and IRF binding sites were predicted. These results demonstrate the conservation of these sites in the immune gene promoter region from oysters to mammals. And in oyster cells, IKKα/β-2 is likely to activate NF-κB and IRF signaling pathway and then regulate the expression of target immune genes.

Research has shown that the Pacific oyster possesses conserved TLR and RLR signaling pathways, and the PRRs, TLR and RLR interact with the downstream adaptor proteins MyD88 and MAVS, respectively, for signaling transduction (34, 35). Co-IP and Y2H assay results revealed that the oyster CgIKKα/β-2 could bind to CgMyD88-1 but did not interact directly with CgMAVS. Further experimental results showed that both CgIKKα/β-1 and CgIKKα/β-2 could interact with oyster TRAF6, which is considered a key ubiquitin E3 mediating TLR and RLR signaling transduction (46, 47). Our previous study also found that the oyster MAVS could interact with TRAF6 (34). In combination with the previous verified signal transduction pathways of RIG-I-MAVS-TRAF6 and TLR-MyD88, the interaction between the oyster IKKα/β-2 and MyD88-1 and between the oyster IKKα/βs and TRAF6 revealed that the IKKα/β proteins of oyster participate in both TLR and RLR signaling, although there is some difference in the transduction of upstream signals. Interestingly, in the results, we noticed that CgIKKα/β-2-flag showed two bands and the band of CgTRAF6 showed some shift. It is reported that, in mammalian cells, phosphorylation of two sites at the activation loop of IKKβ was essential for activation of IKK. And IKKβ auto-phosphorylated at a carboxyl-terminal serine cluster could decreased IKK activity and may prevent prolonged activation (7, 48). Therefore, the two bands of CgIKKα/β-2-flag may be the results of the CgIKKα/β-2 auto-phosphorylation. Regarding the band shift of oyster TRAF6, we noticed that the band shift occurs only when it was co-expressed with CgIKKα/β-2. According to the innate immune signaling transduction of vertebrates, TRAF6 activates IKK by phosphorylating IKK after receiving upstream signal. However, the shift of oyster TRAF6 band may due to the regulation that IKK act on TRAF6 in reverse. It has been reported that in order to prevent excessive immune response and prevent unwanted host damage, the kinase MST4 would directly phosphorylate TRAF6 to limit inflammatory responses (49). Therefore, we hypothesized that the shift of the CgTRAF6 band may be the result of phosphorylation by CgIKKα/β-2. Elucidation of the details and regulation mechanisms of these signaling pathways still require additional in-depth research.

Upon sensing upstream signals, the IKK complex assembly is considered a key step in the activation of downstream transcription factors (50). Human IKKα and IKKβ not only undergo homotypic interactions but also interact with each other (12), and NEMO has been shown to be a crucial scaffold protein and regulatory subunit of the IKK complex. An oyster *NEMO* gene had been cloned in a previous study (39). Therefore, we performed an experiment to examine the relationships among the oyster IKKα/β-1, IKKα/β-2, and NEMO proteins. Our results showed that both the oyster IKKα/β-1 and IKKα/β-2 formed homodimers by interacting with themselves and heterodimers by interacting with each other. Moreover, both the oyster IKKα/β-1 and IKKα/β-2 interacted with the NEMO protein. Thus, we can infer that the oyster IKKα/β-1, IKKα/β-2, and NEMO proteins also form an IKK complex, in which IKKα/β-1 and IKKα/β-2 would be the catalytic subunits and NEMO would act as the scaffold and regulatory subunit.

From the above experimental results, we can speculate that the IKKα/βs of oyster receive upstream signals and form a signal complex; the next question is, how is the immune signal transmitted downward? In other words, how do the IKKα/βs activate NF-κB and ISRE reporter genes? In vertebrates, it has been reported that IKKα can bind to IRFs, resulting in the release of IFN (15). In this study, we studied the interactions between the oyster IKKα/βs and IRFs. Although no IFN homologs have been identified in *C. gigas* so far, several key elements of the IFN pathway are present in the oyster genome, including genes involved in JAK/STAT signaling, IRFs, and many IFN-stimulated genes (ISGs); therefore, it has been hypothesized that the role of IFNs in the oyster may be assumed by novel genes without recognizable homology to vertebrate IFN (36). Our research verified that oyster IKKα/βs could interact with IRF8 and possess the ability to activate ISRE-containing promoters. According to the existing results, we speculate that the oyster IKKα/βs recruit and activate IRF8, which may translocate into the cell nucleus, leading to the activation and expression of “IFN-like” genes. These secreted “IFN-like” cytokines activate the JAK/STAT pathway to stimulate the expression of hundreds of ISGs and initiate an antiviral state in oyster. Additionally, we also observed an interesting observation upon co-expression of CgIKKα/β-1 or CgIKKα/β-2 with IκB proteins, whereby there was an obvious shift in IκB protein band detected upon western blotting. In order to verify whether the shifted bands represent the phosphorylated IκBs, a dephosphorylation assay was performed *in vitro*. And the results showed that shifted bands disappeared after being treated with CIP (Figures 9C,D). Therefore, we speculate that the shifted bands could be the phosphorylated IκBs and oyster IKKα/β-1 or IKKα/β-2 may be able to phosphorylate IκB1 or IκB2. There may be differences when CgIKKα/β-1 or CgIKKα/β-2 phosphorylate CgIκBs. The differential phosphorylation ability of CgIKKα/βs on CgIκB proteins may be a strategy by which the oyster responds to different infections. These hypotheses require further research for validation.

**Figure 10:**
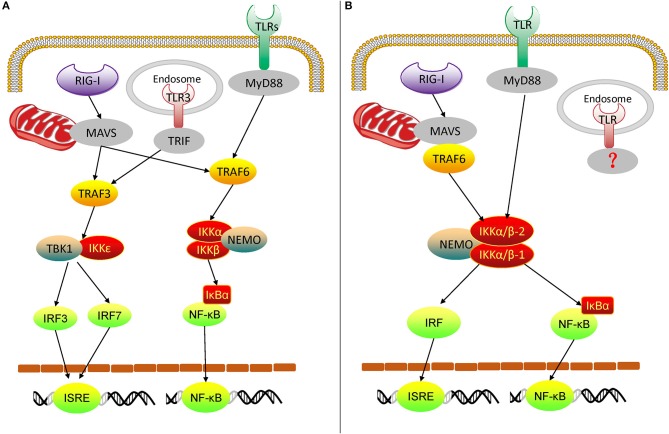
Diagram of vertebrate TLR and RLR signaling **(A)** and oyster TLR and RLR signaling **(B)**. TRIF, TIR-domain-containing adapter inducing interferon-β.

From the above experimental results, we can get an overview of the rudimentary oyster TLR and RLR signaling pathways, mediated by IKKα/β proteins (Figures 10A,B). In the Pacific oyster, IKKα/β proteins are crucial signaling molecules and participate in the crosstalk of oyster TLR and RLR signaling. CgIKKα/β-1 and CgIKKα/β-2 receive the upstream signal from TLRs and RLRs, and then a signaling complex is assembled from CgIKKα/β-1, CgIKKα/β-2, and CgNEMO. After that, in one arm of the pathway, IKKα/βs interact with IRF8 for ISRE activation; in the other arm, IKKα/βs phosphorylate IκB proteins to activate NF-κB. Therefore, oyster IKKα/β proteins play key roles in antiviral and antibacterial innate immune signaling.

In conclusion, we demonstrated that oyster IKKα/βs are versatile innate immune molecules. They participate in the TLR and RLR signaling pathways and mediate signal transduction, followed by the assembly of the IKK complex and activation of NF-κB and IRFs. Our study not only contributes to the understanding of the innate immune mechanisms of invertebrates and the evolution of the vertebrate TLR and RLR signaling pathways, but it also provides a resource for further investigations into oyster immunity and may contribute to the design of novel antiviral or antibacterial strategies for disease control in oysters.

## Data Availability

The raw data supporting the conclusions of this manuscript will be made available by the authors, without undue reservation, to any qualified researcher.

## Ethics Statement

Experiments in this study were conducted with approval from Experimental Animal. Ethics Committee, Institute of Oceanology, Chinese Academy of Sciences, China.

## Author Contributions

BH, LZ, LL, and GZ conceived and designed the experiments and wrote the manuscript. BH, LZ, XT, WW, and ML performed the experiments. FX analyzed the data. All authors read and approved the final manuscript.

### Conflict of Interest Statement

The authors declare that the research was conducted in the absence of any commercial or financial relationships that could be construed as a potential conflict of interest.
