# Supplementary material for: Oyster Versatile IKKα/βs Are Involved in Toll-Like Receptor and RIG-I-Like Receptor Signaling for Innate Immune Response
Source: Front Immunol. 2019 Jul 31;10:1826. doi: 10.3389/fimmu.2019.01826 (PMC6685332; doi:10.3389/fimmu.2019.01826)
Supplement: Supplementary file 1 [file Data_Sheet_1.docx]

**Supplementary Table 1 Primers used in this research**

| **Primer** | **Sequence（5’-3’）** | **Application** |
| --- | --- | --- |
| CgIKKα/β-1-F | ATGGCTCATCCACAGCCAGAGC | Cloning |
| CgIKKα/β-1-R | TCACGGTTCCTTGGATTTCTCTG | Cloning |
| CgIKKα/β-2-F | ATGGCATTTGCACAGCCAA | Cloning |
| CgIKKα/β-2-R | TCACGATGAACTCTCTTTTGCAG | Cloning |
| β-actin-F | GTGCTACGTTGCCCTGGACTT | qRT-PCR |
| β-actin-R | TCGCTCGTTGCCAATGGTGAT | qRT-PCR |
| CgIKKα/β-2-QF | CAAGGAACTGGATCAGGGTAGT | qRT-PCR |
| CgIKKα/β-2-QR | CTTGTGGCAAGAATGGACGGAAT | qRT-PCR |
|  |  |  |
|  |  |  |
| CgIKKα/β-1-myc-F | CATGGAGGCCCGAATTATGGCTCATCCACAGCCAGAGC | Protein expression |
| CgIKKα/β-1-myc-R | CTCGGTCGACCGAATTTCACGGTTCCTTGGATTTCTCTG | Protein expression |
| CgIKKα/β-2-myc-F | CATGGAGGCCCGAATTATGGCATTTGCACAGCCAA | Protein expression |
| CgIKKα/β-2-myc-R | CTCGGTCGACCGAATTTCACGATGAACTCTCTTTTGCAG | Protein expression |
| CgIKKα/β-1-flag-F | CTCCATATGACTAGTCTCGAGATGGCTCATCCACAGCCAGAGC | Protein expression |
| CgIKKα/β-1-flag-R | TACCACGCGTGAATTCTCGAGTCACGGTTCCTTGGATTTCTCTG | Protein expression |
| CgIKKα/β-2-flag-F | CTCCATATGACTAGTCTCGAGATGGCATTTGCACAGCCAA | Protein expression |
| CgIKKα/β-2-flag-R | TACCACGCGTGAATTCTCGAGTCACGATGAACTCTCTTTTGCAG | Protein expression |
| CgMyD88-1-myc-F | CATGGAGGCCCGAATTATGTCGATCACATCGGAACAGT | Protein expression |
| CgMyD88-1-myc-R | CTCGGTCGACCGAATTTCAGCCGTTGTATGGAGTGTTGTC | Protein expression |
| CgTRAF6-myc-F | CATGGAGGCCCGAATTATGGGGACGTCAGAGCGACCTTTG | Protein expression |
| CgTRAF6-myc-R | CTCGGTCGACCGAATTTCACTCCGATCTCCCAATGATCTGA | Protein expression |
| CgNEMO-myc-F | CATGGAGGCCCGAATTATGAATGGAGCCCACACGC | Protein expression |
| CgNEMO-myc-R | CTCGGTCGACCGAATTTCAGGTCATGTCCTGGTCGATA | Protein expression |
| CgIRF-8-myc-F | CATGGAGGCCCGAATTATGGCAACAGAAATTGATATTCGCAG | Protein expression |
| CgIRF-8-myc-R | CTCGGTCGACCGAATTTCAGGTTTCCATTTGTCCATGGCAT | Protein expression |
| CgIκB1-myc-F | CATGGAGGCCCGAATTATGTCGAACAGAGACTTTGCTCG | Protein expression |
| CgIκB1-myc-R | CTCGGTCGACCGAATTTCAACTCATATCTTCCTCACTATCT | Protein expression |
| CgIκB2-myc-F | CATGGAGGCCCGAATTATGGATTTGAACGACCTGGAAG | Protein expression |
| CgIκB2-myc-R | CTCGGTCGACCGAATTTCAATCTTCTTCCTCCTCCGATTC | Protein expression |
| CgIκB3-myc-F | CATGGAGGCCCGAATTATGGCATTTCGGAAACCAGC | Protein expression |
| CgIκB3-myc-R | CTCGGTCGACCGAATTTTAAGTGTCGTCAATTTCTTCACG | Protein expression |
|  |  |  |
|  |  |  |
| CgIKKα/β-1-AD-F | GGAGGCCAGTGAATTCATGGCTCATCCACAGCCAGAGC | Y2H |
| CgIKKα/β-1-AD-R | CGAGCTCGATGGATCCCGGTTCCTTGGATTTCTCA | Y2H |
| CgIKKα/β-2-AD-F | GGAGGCCAGTGAATTCATGGCATTTGCACAGCCAA | Y2H |
| CgIKKα/β-2-AD-R | CGAGCTCGATGGATCCCGATGAACTCTCTTTTGCAG | Y2H |
| CgMyD88-1-BD-F | CATGGAGGCCGAATTCATGTCGATCACATCGGAACAGT | Y2H |
| CgMyD88-1-BD-R | GCAGGTCGACGGATCCGCCGTTGTATGGAGTGTTGTCC | Y2H |
| CgTRAF6-AD-F | GGAGGCCAGTGAATTCATGGGGACGTCAGAGCGACCTTTG | Y2H |
| CgTRAF6-AD-R | CGAGCTCGATGGATCCTCACTCCGATCTCCCAATGATCTGA | Y2H |
| CgIKKα/β-2-BD-F | CATGGAGGCCGAATTCATGGCATTTGCACAGCCAA | Y2H |
| CgIKKα/β-2-BD-R | GCAGGTCGACGGATCCCGATGAACTCTCTTTTGCAG | Y2H |
| CgNEMO-BD-F | CATGGAGGCCGAATTCATGAATGGAGCCCACACGC | Y2H |
| CgNEMO-BD-R | GCAGGTCGACGGATCCTCAGGTCATGTCCTGGTCGATA | Y2H |
| CgIRF8-AD-F | GGAGGCCAGTGAATTCATGGCAACAGAAATTGATATTCGCAG | Y2H |
| CgIRF8-AD-R | CGAGCTCGATGGATCCTCAGGTTTCCATTTGTCCATGGCAT | Y2H |


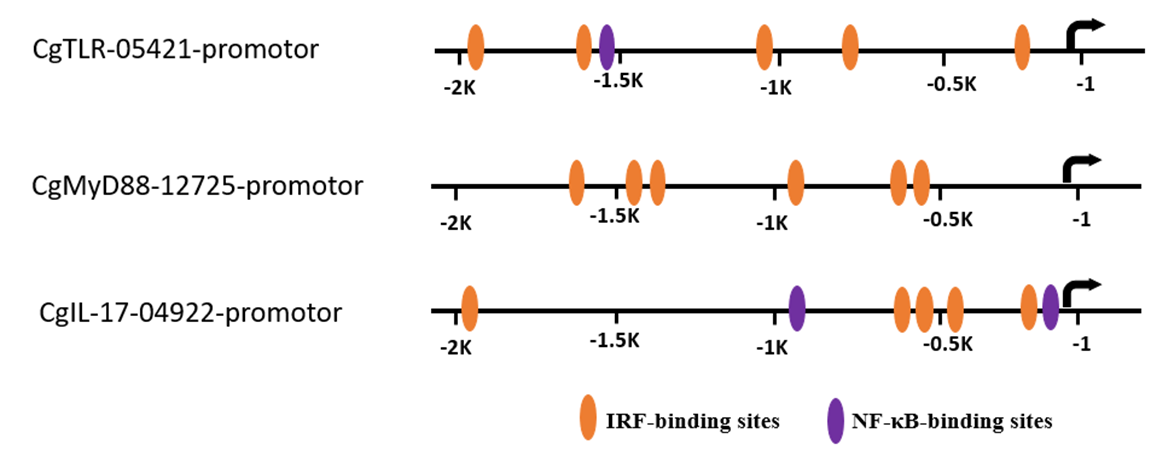


**Supplementary Figure 1** The predicted NF-κB and IRF binding sites of some oyster immune genes promoter. The genome sequence was obtained from the published oyster database (http://oysterdb.

cn/). And Potential transcriptional factor binding sites in the promoter region were analyzed using the TRANSFAC software (<http://www.gene-regulation.com/pub/programs.html>).


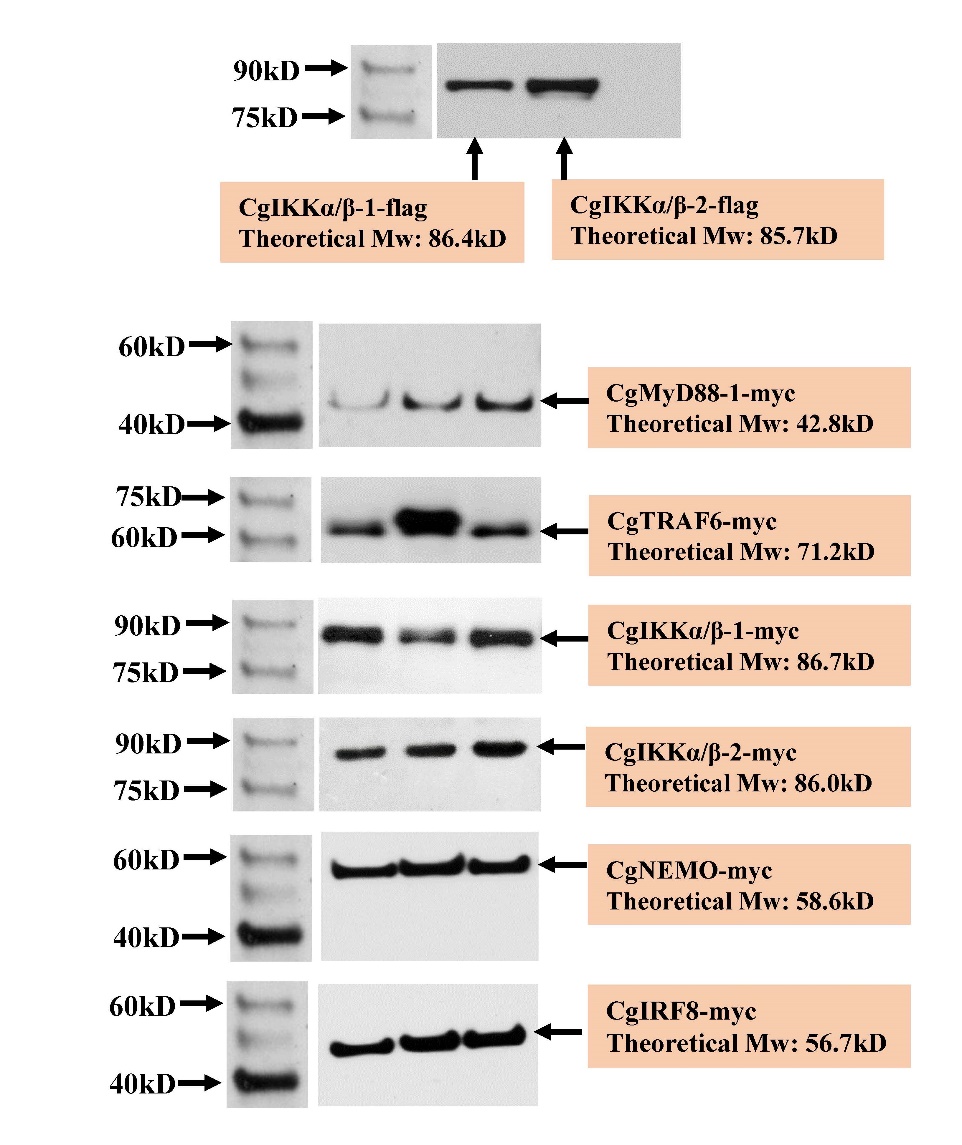


**Supplementary Figure 2** Theoretical molecular weight and specificity of proteins detected for Western Blot analysis in this research


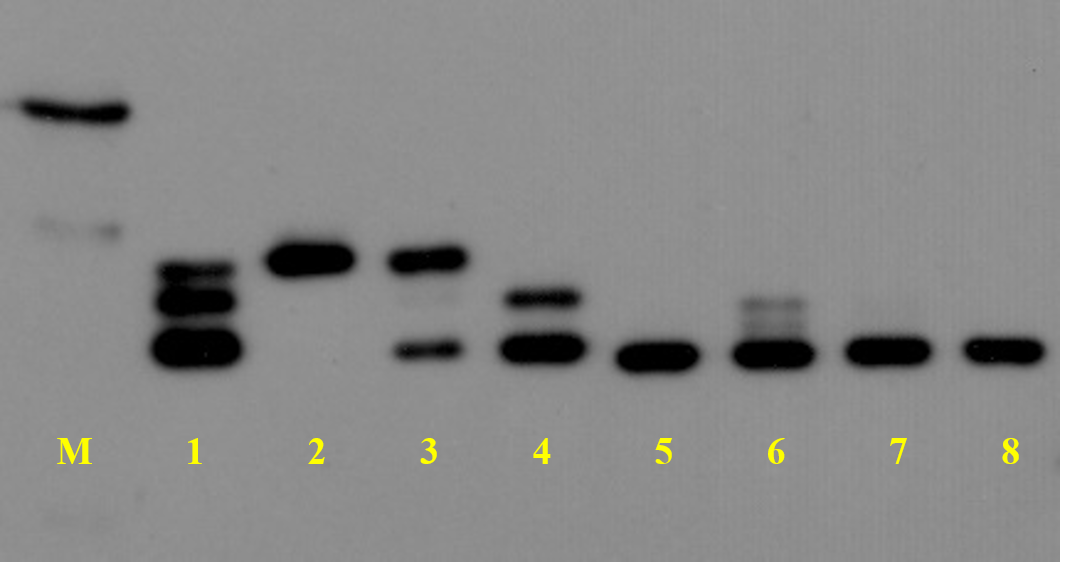


**Supplementary Figure 3** CgIKKα/β-1, CgIKKα/β-2 and CgIKKε-like expression vectors were co-transfected with oyster CgIκB1 and CgIκB2 protein vectors. M: Western blotting protein marker; lanes 1–4 show CgIκB1 protein bands when co-transfected with CgIKKα/β-1, CgIKKα/β-2, CgIKKε-like and empty control vector. Lanes 5–8 show CgIκB2 protein bands when co-transfected with CgIKKα/β-1, CgIKKα/β-2, CgIKKε-like and empty control vector. (CgIκB1 and CgIκB2 protein bands when co-transfected with CgIKKε-like was not shown in the manuscript and the relation between CgIκB proteins and CgIKKε-like will be discussed in another research.)
